# Essential Oils From *Citrus unshiu* Marc. Effectively Kill *Aeromonas hydrophila* by Destroying Cell Membrane Integrity, Influencing Cell Potential, and Leaking Intracellular Substances

**DOI:** 10.3389/fmicb.2022.869953

**Published:** 2022-06-28

**Authors:** Weiming Zhong, Kangyong Chen, Linlin Yang, Tao Tang, Sifan Jiang, Jiajing Guo, Zhipeng Gao

**Affiliations:** ^1^Hunan Engineering Technology Research Center of Featured Aquatic Resources Utilization, College of Animal Science and Technology, Hunan Agricultural University, Changsha, China; ^2^Hunan Agriculture Product Processing Institute, Hunan Academy of Agricultural Sciences, Changsha, China

**Keywords:** *Citrus unshiu* Marc. essential oil, chemical composition, antibacterial activity, *Aeromonas hydrophila*, mode of action

## Abstract

*Aeromonas hydrophila* is one of the important pathogenic bacteria in aquaculture causing serious losses every year. Essential oils are usually used as natural antimicrobial agents to reduce or replace the use of antibiotics. The aim of this study was to evaluate the antibacterial activity and explore the mechanisms of essential oil from satsuma mandarin (*Citrus unshiu* Marc.) (SMEO) against *A. hydrophila*. The results of the gas chromatography-mass spectrometer demonstrated that SMEO contains 79 chemical components with the highest proportion of limonene (70.22%). SMEO exhibited strong antibacterial activity against *A. hydrophila in vitro*, the diameter of the inhibition zone was 31.22 ± 0.46 mm, and the MIC and MBC values were all 1% (v/v). Intracellular material release, scanning electron microscopy (SEM), transmission electron microscopy (TEM), and flow cytometry analysis revealed the dynamic antibacterial process of SMEO, the morphological changes of bacterial cells, and the leakage process of intracellular components. These results demonstrated that SMEO disrupted the extracellular membrane permeability. Our study demonstrated that SEMO has the potential to be used to control and prevent *A. hydrophila* infections in aquaculture.

## Introduction

In recent years, with the rapid development and expansion of the aquaculture industry in the world, outbreaks of diseases in aquatic animals are increasing, which have become serious threats to the sustainable development of aquaculture. Compared with other pathogens (e.g., viruses, fungi, and parasites), diseases caused by bacteria have become a major obstacle to aquaculture, because bacteria can survive independently in the aquatic environment without the presence of a host. Among these bacterial pathogens, diseases caused by *Aeromonas hydrophila* have caused huge economic losses to aquaculture in the world (Vivekanandhan et al., 2002; Rico et al., 2013; Stratev and Odeyemi, 2017). *A. hydrophila* belongs to Vibrionaceae and *Aeromonas* genus, which is a type strain of *Aeromonas* (Hamid et al., 2016). It is an opportunistic pathogenic bacterium with a wide range of hosts, including fish, mollusks, crustaceans, amphibians, reptiles, poultry, mammals, and humans (Janda and Abbott, 2010; Parker and Shaw, 2011). *A. hydrophila* is widely present in water, soil, silt and biological body surfaces, digestive tracts, and feces (Yardimci and Aydin, 2011; Rasmussen-Ivey et al., 2016; Fernández-Bravo and Figueras, 2020). The main symptoms of infection include local damage, necrosis, surface hemorrhage, edema, and abdominal distension (Sapkota et al., 2008; Rico and Van den Brink, 2014). At present, the methods for the control and prevention of *A. hydrophila* in aquaculture mainly include medical, immune, and biological therapies. Indeed, medical therapies, especially antibiotics, are still the main method used for the prevention of *A. hydrophila*.

However, the abuse or overuse of antibiotics is widespread in aquaculture, which increases the resistance of microorganisms (antimicrobial resistance, AMR), and ultimately leads to the emergence of drug-resistant microorganisms (Yang et al., 2019). Several studies have shown that the abuse or overuse of antibiotics [such as tetracycline (Nawaz et al., 2006), enrofloxacin (Zhu et al., 2017), ampicillin (Erdem et al., 2010)] increased the drug resistance of *A. hydrophila*. Meanwhile, multiple drug-resistant strains of *A. hydrophila* were found in a different variety of fishes, which may enter the food chain through aquatic products and infect humans (Vivekanandhan et al., 2005). Moreover, the overuse of antibiotics may also cause antibiotic residues in aquatic animals and the environment, which is a serious threat to food safety and human health (Majolo et al., 2017; Guo et al., 2018; Gao et al., 2020).

Over the years, the problems caused by drug-resistant microorganisms have caused widespread concern around the world. Many countries have issued the “National Action Plan for Antimicrobial Resistance (Shallcross and Davies, 2014),” which aimed to deal with the risks and challenges posed by microbial resistance. One of the main measures in the “Plan” pointed out that the development of “antibiotic alternatives” is an important way to solve the problem of drug resistance. Essential oils (EOs), one of the promising antibiotic alternatives, is a kind of volatile oily liquid substance extracted from different parts of a plant, such as fruits, seeds, peels, flowers, and leaves (El Asbahani et al., 2015). Many investigations indicated that EOs have antimicrobial, insecticidal, antioxidant, and anti-inflammatory activities (Bakkali et al., 2008). When focusing on the antimicrobial part, EOs are widely used as human drugs, veterinary drugs, and food preservatives (Idris et al., 2017; Trifan et al., 2020). But until now, the studies focusing on the antimicrobial activity of EOs in aquaculture are very limited.

China is the world’s largest producer of citrus. In addition to fresh food, citrus is mainly used for processing (such as juice and canned food), but this processing may produce a mass of citrus peels (Gómez-Mejía et al., 2019). Extracting EO from the peels is a promising way for its comprehensive utilization. Thus, in this study, EO was extracted from one of the major citrus cultivars in China, Satsuma mandarin (*Citrus unshiu* Marc.). Furthermore, its antibacterial activity and mechanism of action against *A. hydrophila* were investigated. We aimed to develop a natural antibiotic alternative (EO) to solve the problems of drug resistance and antibiotic residues and provide a new method for the prevention and treatment of *A. hydrophila* in aquaculture.

## Materials and Methods

### Microorganism and Culture Conditions

*Aeromonas hydrophila* (PRJNA808687) used in this study was from our lab, which was isolated from grass carp (*Ctenopharyngodon Idella*) in 2018. *A. hydrophil* (CICC25017) was purchased from the China Center of Industrial Culture Collection. *A. hydrophila* was streaked on Tryptone Soy Agar (TSA, Guangdong Huankai Microbial Sci. & Tech. Co., Ltd., China) and incubated at 28°C for 24 h, and then 5 colonies were transferred to 5 ml of Tryptone Soy Broth (TSB, Guangdong Huankai Microbial Sci. & Tech. Co., Ltd., China) and incubated for 8 h at 28°C with shaking.

### Steam Distillation of Essential Oil From Satsuma Mandarin

Satsuma mandarin was collected from Neijiang city, Sichuan Province, China. In the Clevenger apparatus, 500 g of satsuma mandarin peels were suspended in 2,000 ml of distilled water and subjected to steam distillation. The extraction was carried out for 3 h, and the obtained EO was collected, dried by anhydrous sodium sulfate (Na_2_SO_4_) for 24 h, and then stored at 4°C in brown glass vials.

### Gas Chromatography-Mass Spectrometry

The GC-MS analysis was carried out by using an Agilent 7890A GC equipped with a Gerstel MPS autosampler, coupled with an Agilent 5975C MSD detector. The chromatographic separation was performed on an HP-5MS capillary column 30 m × 0.25 mm i.d., 0.25 μm, the GC oven was operated at 40°C held for 1 min, increased to 220°C at a rate of 3°C/min, held at 220°C for 25 min, increased to 250°C at a rate of 5°C/min, and finally held for 10 min. Helium was used as the carrier gas at a flow rate of 1 ml/min. The injector and detector temperatures were set at 250 and 280°C, respectively. The mass spectrometer was operated in the 70 eV EI mode with scanning from 35 to 350 amu/s, and the ion source was set at 230°C. The EO components were identified by matching their recorded mass spectra with the data bank NIST 08 (National Institute of Standards and Technology).

### Agar Diffusion Method

A volume of 100 μl bacterial dilution (PRJNA808687 and CICC25017, 1 × 10^6^ CFU/ml) was evenly smeared on Mueller-Hinton Agar (MHA). Following that, filter paper disks (6 mm in diameter) containing 6 μl SMEO were placed on the surface of the agar plates. After standing for 10 min, the plates were incubated at 28°C for 12 h. Meanwhile, antibiotics (florfenicol and amoxicillin) and water + 1% Tween were used as positive and negative controls, respectively. Finally, the diameters of the inhibition zone (DIZ) were measured (Oonmetta-aree et al., 2006).

### Determination of Minimum Inhibitory Concentration and Minimum Bactericidal Concentration

Serial dilutions of SMEO ranging from 0.0625 to 16% were prepared in TSB (with 1% Tween 20). A volume of 100 μl of each SMEO serial dilution was dispensed into 96 microtiter plates, respectively. Afterward, 100 μl of the bacterial suspension (PRJNA808687, 2 × 10^6^ CFU/ml) was treated with the dilutions of SMEO. Then, the microtiter plates were cultured at 28°C for 120 rpm. After 24 h incubation of the bacterial suspension, the lowest concentration of SMEO with no bacterial growth was determined as MIC. Furthermore, to determine MBC, 10 μl of the above solutions (from the concentration of MIC to 16%) were placed on TSA plates and incubated at 28°C for 24 h. MBC was the lowest concentration of SMEO without any visible colonies on the plates.

### Bacterial Growth Kinetics

Bacterial growth kinetics was measured by the effect of SMEO on bacterial growth (Sureshkumar et al., 2010). According to the above experimental data, 0 × MIC, 0.0625 × MIC, 0.125 × MIC, 0.25 × MIC, 0.5 × MIC, and 1 × MIC of SMEO were diluted with 40 ml bacterial suspension (PRJNA808687, 1 × 10^6^ CFU/ml). The suspensions were incubated at 28°C for shaking (120 rpm), and the absorbance was measured by a spectrophotometer (UV759S, INESA Analytical Instrument Co., Ltd., China) at an optical density of 600 nm at the following time points (t/h): 0, 2, 4, 6, 8, 10, 12, 14, 16, 18, 20, 22, 24, 26, 28, 30, 36, and 48 h.

### Leakage of Cellular Components Assay

A total of 3–4 colonies of *A. hydrophila* were transferred into 10 ml TSB and incubated at 28°C overnight. After incubation, cells were collected by centrifugation at 8,000 rpm for 10 min and adjusted to the concentration of 1 × 10^9^ CFU/ml (PRJNA808687) in 10 ml of phosphate-buffered solution (PBS). Then, SMEO at the concentration of 1 × MIC was added to the abovementioned solutions and treated at 28°C for 4, 8, and 12 h. Finally, the supernatants were separated from bacterial cells by centrifugation (8,000 rpm) and filtration (with 0.22 μm filter, Sigma-Aldrich, United States), which will be used for the nucleic acid and protein assay as described. At the same time, SMEO treatment for 0 h was used as the zeroing sample for UV spectroscopic detection.

For the nucleic acid assay, the absorbance of the supernatant samples was measured by a spectrophotometer (UV759S, INESA Analytical Instrument Co., Ltd., China) at the optical density of 260 nm.

For the protein assay, a Total Protein Kit, Micro (Sigma-Aldrich, United States) was used to measure the concentration of protein. According to the operating instructions, the protein assay solution was mixed with the supernatant samples. After approximately 2 min, the absorbance of the sample was also measured by a spectrophotometer at the optical density of 595 nm (UV759S, INESA Analytical Instrument Co., Ltd., China).

### Scanning Electron Microscope and Transmission Electron Microscope

*Aeromonas hydrophila* (PRJNA808687) was incubated to log phase as the conditions mentioned in the “Microorganism and culture conditions” section; after incubation, bacterial suspensions were adjusted to 1 × 10^8^ CFU/ml. Cells (2 ml suspensions) were collected by centrifugation at 8,000 rpm for 10 min, and the bacterial pellet was carefully washed with PBS and resuspended. Later, 1 × MIC SMEO (with 1% Tween 20) was added to the resuspension and treated for 4, 8, and 12 h. Finally, the samples were collected by centrifugation, washed three times with PBS, and fixed with 2.5% glutaraldehyde at 4°C for 4 h or overnight. Next, the samples were prepared for SEM and TEM assay as described.

For SEM, the glutaraldehyde solution was removed and the bacteria samples were washed with PBS for three times, and then dehydrated with 10, 30, 50, 70, and 90% ethanol; in turn, 15 min each time, and dehydrated two times with 100% ethanol, 20 min each time. After dehydration, ethanol was replaced with tertiary butanol two times for 20 min each, then the samples were vacuum freeze-dried, sprayed with gold, and observed by scanning electron microscope (Hitachi S-4800, Hitachi, Japan).

For TEM, the fixed solution was removed, and the samples were washed three times with PBS, then the bacteria samples were placed on the copper mesh, air-dried for 5 min, and negatively stained with 1% phosphotungstic acid. After that, the samples were observed by a transmission electron microscope (Hitachi HT-7700, Hitachi, Japan).

### Flow Cytometry Analysis

Flow cytometry analysis was carried out to investigate the effects of SMEO on *A. hydrophila* (PRJNA808687). The method for SMEO treatment was the same as mentioned in the “Leakage of Cellular Components Assay” section, but the treatment time is 8 h. Meanwhile, PBS and ethanol treatment were used as negative and positive controls, respectively. After treatment, bacterial cells were collected by centrifugation and adjusted to the concentration of 1 × 10^6^ CFU/ml; then different staining procedures proceeded as follows.

### Membrane Permeability [Thiazole Orange and Propidium Iodide Staining]

Thiazole orange (TO, Sigma-Aldrich, United States) and propidium iodide (PI, Sigma-Aldrich, United States) were used to evaluate the membrane permeability of cells. For TO staining, 1-μl TO solution was added to 1-ml bacterial suspensions (final concentration of TO: 10 μg/ml in DMSO), and then incubated at room temperature for 15 min. For PI staining, 1-μg PI was added to 1 ml bacterial suspensions (final concentration of PI: 1 μg/ml in PBS), and then incubated at 37°C for 15 min.

### Membrane Potential [Bis-1,3-Dibutylbarbutiric Acid (BOX) and Propidium Iodide Staining]

Bis-1,3-dibutylbarbutiric acid (BOX, Sigma-Aldrich, United States) and PI were used to evaluate the membrane potential of cells. For BOX staining, 2.5-μg BOX was added to 1-ml bacterial suspensions (final concentration of BOX: 2.5 μg/ml in PBS with 4 mM EDTA), and then incubated at 37°C for 15 min. For PI staining, the procedure was the same as mentioned in the “Membrane Permeability (TO and PI Staining)” section.

### Efflux Activity [Ethidium Bromide Staining]

Ethidium bromide (EB, Sigma-Aldrich, United States) was used to evaluate the efflux activity of cells. For EB staining, 10-μg EB was added to 1-ml bacterial suspensions (final concentration of EB: 10 μg/ml in DMSO) and then incubated at 37°C for 15 min.

After finishing these staining procedures, samples were washed three times with PBS, and the concentration of bacterial suspensions was adjusted to about OD_600_ = 0.1. Then, the samples were placed on ice for flow cytometry analysis by a flow cytometer (BD Accuri C6 plus, BD, United States), green fluorescence was collected in the FL1 channel (533 nm), and red fluorescence in the FL3 channel (>670 nm). Fluorescence signals were collected by FL1 (TO and BOX) and FL3 (PI, EB) bandpass filters. Bacterial cells were gated per the FSC/SSC parameters and a total of 5,000 events were acquired for each sample.

### Statistical Analysis

All the experiments were performed in triplicate. Statistical analysis was carried out using SPSS and GraphPad Prism 7 for the *t*-tests. All asterisks indicate significant differences (*p* < 0.05).

## Results and Discussion

### The Chemical Composition of Essential Oil From Satsuma Mandarin

The chemical composition of SMEO is summarized in Table 1. A total of 79 components were identified, which accounted for the percentage of 99.9997%. Meanwhile, 96.3238% of SEMO constituents were monoterpenes. Limonene was the most abundant component with a percentage of 70.2252%, followed by γ-terpinene (7.8955%), β-myrcene (5.0086%), l-α-pinene (3.8957%), β-terpinene (1.7055%), linalool (1.5054%), and α-terpineol (1.0265%); these seven components account for 91.2624% of all the components. The percentage of the other remaining 72 components was below 1% (0.0097–0.8700%).

**TABLE 1:** Chemical composition of SMEO.

Num	Cas	Ingredient	Percentage (%)
————————————————-monoterpene alkenes—————————————–			
1	000138-86-3	Limonene	70.2479
2	000099-85-4	γ-Terpinene	7.8955
3	000123-35-3	β-Myrcene	5.0086
4	000123-35-3	L-α-Pinene	3.8957
5	000099-84-3	β-Terpinene	1.7055
6	000586-62-9	Terpinolene	0.87
7	005293-90-3	Cyclohexene, 2-ethenyl-1,3,3-trimethyl-	0.1578
8	058037-87-9	4-Methyl-1-(1-methylethyl)bicyclo[3.1.0]hexane didehydro deriv.	0.1252
9	002792-39-4	2,6-Octadiene, 2,6-dimethyl-	0.1097
10	013837-95-1	Cyclohexane,1-methylene-3-(1-methylethenyl)-, (3R)-	0.0923
11	000471-84-1	α-Fenchene	0.0758
12	018680-59-6	3(7)-Carene, 4- hydroxymethyl-, exo-	0.0568
13	005256-65-5	Cyclohexene, 3-methyl-6-(1-methylethyl)-	0.0410
Total			90.2818
———————————————monoterpene alcohols——————————————–			
1	000078-70-6	Linalool	1.5054
2	000098-55-5	α-Terpineol	1.0265
3	020126-76-5	L-terpinen-4-ol	0.5497
4	000106-22-9	Citronellol	0.262
5	001946-00-5	Limonene glycol	0.2304
6	001197-06-4	(Z)-Carveol	0.1901
7	000138-87-4	β-Terpineol	0.1206
8	018881-04-4	Verbenol	0.0955
9	015358-81-3	o-Mentha-1(7),8-dien-3-ol	0.0443
10	000106-24-1	geraniol	0.0360
Total			4.0605
———————————————–monoterpene ethers———————————————			
1	006909-30-4	(+)-(E)-Limonene oxide	0.3637
2	001076-56-8	Thymol methyl ether	0.3635
3	004680-24-4	Limonene oxide	0.3102
Total			1.0374
———————————————monoterpene aldehydes——————————————			
1	002385-77-5	(R)-(+)-citronellal	0.1731
2	002111-75-3	Perillal	0.1603
3	002363-88-4	2,4-Decadienal	0.1232
Total			0.4566
———————————————-monoterpene ketones——————————————-			
1	002244-16-8	D-Carvone	0.1033
2	002520-60-7	Cyclopentanone,2-(3-methyl-2-buten-1-yl)-	0.0778
3	000089-81-6	Piperitone	0.0255
4	000076-22-2	Camphor	0.0214
Total			0.2280
———————————————–monoterpene others——————————————–			
1	000141-12-8	Neryl Acetate	0.1528
2	1000149-84-5	Myrcenylacetat	0.0465
3	031076-73-0	Trifluoroacetyl-.alpha.-fenchol	0.0433
4	014049-11-7	Epoxylinalool	0.0169
Total			0.2595
————————————————sesquiterpene alkenes—————————————–			
1	017699-05-7	α-Bergamotene	0.1622
2	026560-14-5	(Z,E)-α-Farnesene	0.1501
3	033880-83-0	β-elemene	0.1387
4	000502-61-4	α-Farnesene	0.1162
5	020307-84-0	δ-Elemene	0.0894
6	339154-91-5	γ-Elemene	0.0867
7	023986-74-5	Germacrene D	0.0856
8	1000156-82-4	Cycloundeca-2,6,9-triene, 1,1,5,9-tetramethyl-	0.0592
9	1000062-61-9	1,4,7,-Cycloundecatriene, 1,5,9,9- tetramethyl-, Z,Z,Z-	0.0579
10	000483-76-1	(+)-δ-cadinene	0.048
11	339154-91-5	γ-Elemene	0.0308
12	028580-43-0	Ledane	0.0306
13	018794-84-8	β-Farnesene	0.0263
14	003691-12-1	α-Guaiene	0.0097
Total			1.0914
——————————————–sesquiterpene alcohols——————————————-			
1	161362-94-3	7R,8R-8-Hydroxy-4-isopropylidene-7-methylbicyclo[5.3.1]undec-1-ene	0.0276
2	007212-44-4	Nerolidol	0.0241
Total			0.0517
———————————————-sesquiterpene ethers——————————————–			
1	1000159-36-6	Isoaromadendrene epoxide	0.1386
——————————————–sesquiterpene aldehydes——————————————			
1	017909-77-2	α-sinensal	0.0884
——————————————— sesquiterpene ketones——————————————-			
1	1000164-02-7	Bicyclo[6.3.0]undec-1(8)-en-3-one, 2,2,5,5-tetramethyl-	0.0296
——————————————————-others—————————————————–			
1	000112-31-2	Decanal	0.7489
2	002511-91-3	1-Cyclopropylpentane	0.2388
3	077899-10-6	14-Tricosen-1-ol,1-formate, (14Z)-	0.221
4	109746-13-6	1,4-Methanophthalazine, 1,4,4a,5,6,7,8,8a-octahydro-9,9- dimethyl-, (1alpha,4alpha,4aalpha,8aalpha)-	0.1936
5	1000221-94-3	Benzylidene-(3,4-methylendioxy),-N,N’-heptane-(1,7-diamino[bis-	0.1202
6	005164-65-8	2-Methylenebicyclo[2.1.1]hexane	0.1063
7	001759-64-4	Cyclohexene,1,6-dimethyl-	0.0956
8	000334-48-5	Decanoic acid	0.0617
9	007206-15-7	(E)-4-Dodecene	0.0601
10	1000336-51-0	Methyl 5,13-docosadienoate	0.0582
11	054889-48-4	1,1-Diethoxyoctane	0.0522
12	002497-25-8	2-Decenal, (Z)-	0.0503
13	000057-10-3	Palmitic acid	0.0482
14	005353-25-3	Emulphor	0.0466
15	000292-64-8	Cyclooctane	0.0463
16	086711-81-1	Hexadecyl 2-chloropropanoate	0.0275
17	000544-12-7	3-Hexen-1-ol	0.0271
18	1000345-15-3	Fumaric acid, di(cyclohex-3-enylmethyl) ester	0.018
19	038061-92-6	2-Methyl-oct-2-enedial	0.0167
20	1000282-85-6	2,2-Dimethylpropanoic acid, heptadecyl ester	0.0156
21	000505-57-7	2-Hexenal	0.0128
22	034756-98-4	Portulol	0.0105
Total			2.2762
Total monoterpenes compounds			96.3238
Total sesquiterpenes compounds			1.3997

### The Antibacterial Activity of SMEO

**FIGURE 1:**
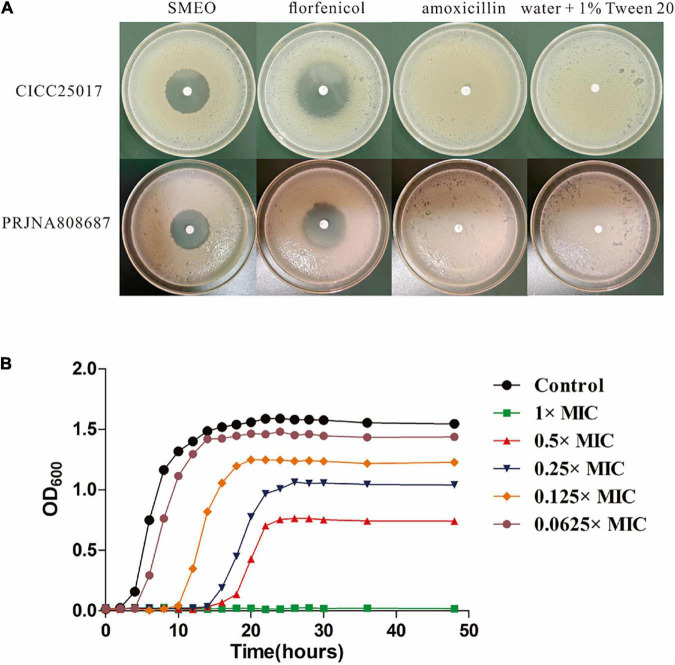
The antibacterial activity of SMEO against *Aeromonas hydrophila*. **(A)** The diameter zone of inhibition (mm). **(B)** *A. hydrophila* growth curves with or without different concentrations of SMEO.

As shown in Figure 1A and Table 2, SMEO florfenicol exhibited strong antibacterial activity against *A. hydrophila in vitro* (PRJNA808687 and CICC25017). Among them, the inhibition diameter of SEMO against *A. hydrophila* was 31.22 ± 0.46 mm. The bacteria were completely killed when the SEMO concentration was 1%. During bacterial growth period, *A. hydrophila* was exposed to SEMO to confirm whether the environmental adaptations of cells were affected. The antibacterial kinetic curves of SMEO were demonstrated in Figure 1B, which reflected the kinetic character of SMEO at different concentrations (0.0625 × MIC, 0.125 × MIC, 0.25MIC, 0.5 × MIC, and 1 × MIC) and treated times. In the 0.0625 × MIC, 0.125 × MIC, 0.25 × MIC, and 0.5 × MIC groups, the increasing lag phase time and decreasing concentration of bacteria during the stationary phase indicated that the cells were more sensitive to the environmental stress in which SMEO was located. Meantime, bacterial growth was not observed at the concentration of 1 × MIC.

**TABLE 2:** The antibacterial activity of different drugs.

Name of drug	Bacterial strain	Inhibition zone(mm)	MIC(v/v)	MBC(v/v)
SMEO	CICC25017	30.82 ± 0.56		
	PRJNA808687	31.22 ± 0.46	1%	1%
Florfenicol	CICC25017	33.76 ± 0.55		
	PRJNA808687	33.34 ± 3.58		
Amoxicillin	CICC25017	–		
	PRJNA808687	–		
Water + 1% Tween 20	CICC25017	–		
	PRJNA808687	–		

*Note: Highly sensitive (d > 18 mm), moderately sensitive (10 mm < d ≤ 18 mm), and low or no sensitivity (d ≤ 10 mm). - represents a drug-sensitive diameter of 6 mm.*

### The Release of Intracellular Material

**FIGURE 2:**
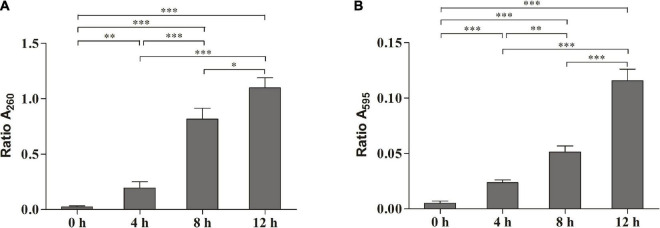
The release of the intracellular material of *A. hydrophila* at different treatment times (0, 4, 8, and 12 h) in 1 × minimum inhibitory concentration (MIC) SMEO. **(A)** Change of the optical absorbance (A_260_) of cell supernatant, which is an indicator of nucleic acids. **(B)** Change of the optical absorbance (A_280_) of cell supernatant, which is an indicator of protein. ^∗^, ^∗∗^, and *** represent *P* < 0.05, *P* < 0.01, and *P* < 0.001.

The release of the bacterial intracellular material was observed by measuring changes in the composition of cell supernatant. Figure 2A shows the changes in nucleic acids. When the cells were exposed to SMEO at the concentrations of 2 × MIC for 4, 8, and 12 h, the values of A_260_ were 0.1950 ± 0.0324, 0.8163 ± 0.0573, and 1.0992 ± 0.0529, respectively. The results demonstrated that the concentration of nucleic acids in the supernatant increased with the increase of exposure time. Figure 2B shows the protein changes by Coomassie blue staining, which indicated that the concentration of proteins increased significantly (*p* < 0.05) with the increase of exposure time, with values of 0.0237 ± 0.0015, 0.0513 ± 0.0032, and 0.1157 ± 0.0061 in A_595_, respectively. Meanwhile, the changes in protein and nucleic acid concentrations in the extracellular supernatant demonstrate that SMEO was able to rupture bacterial cell membranes.

### Scanning Electron Microscopy Observations

**FIGURE 3:**
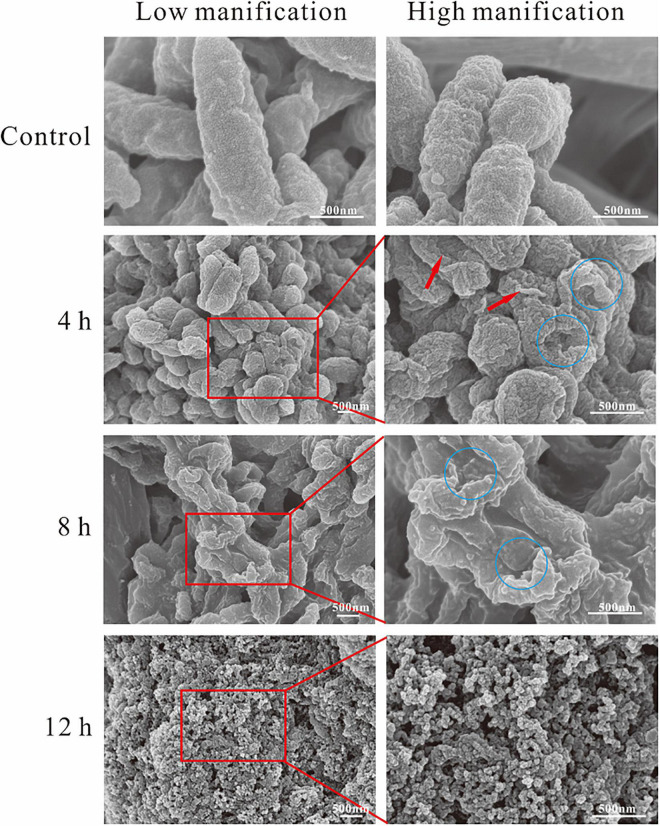
Scanning electron microscopy (SEM) photography of *A. hydrophila* planktonic cell. (Control) Untreated group; (6, 12, and 18 h) bacteria treated by SMEO at 1 × MIC for 6, 12, and 18 h, respectively. The red arrows and blue circles indicate wrinkled surfaces and cell collapse.

**FIGURE 4:**
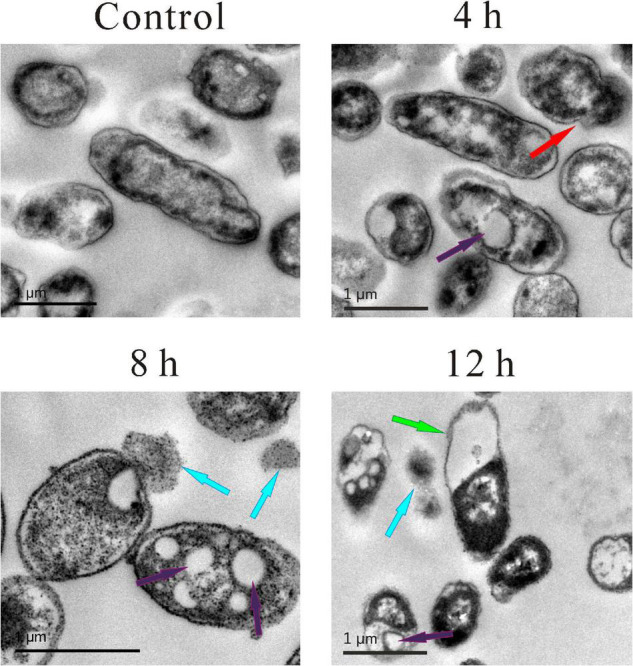
Transmission electron microscopy (TEM) photography of *A. hydrophila* planktonic cell. (Control) Untreated group; (6, 12, 18 h) bacteria treated by SMEO at 1 × MIC for 6, 12, and 18 h, respectively. The red, purple, blue, and green arrows indicate wrinkled surfaces, vacuoles inside the cytoplasm, the leakage of cytoplasm, and the cell wall without cytoplasm inside, respectively.

Scanning electron microscopy was used to reveal the changes in themorphology of *A. hydrophila* with and without the treatment of SMEO. As shown in Figure 3, cells in the control group were rod-shaped with smooth surfaces and intact structures, about 1–2 μm in length. Compared with the control group, the morphology of cells in those three experimental groups showed significant changes, wrinkled surfaces (pointed by the red arrow, Figure 3, 4h), and collapsed cells (pointed by the blue circle, Figure 3, 4,8h), indicating the huge damage caused by SMEO. The degree of SMEO-induced morphological changes and the quantity of affected cells enhanced with the increase in treatment time. First, after a 6-h treatment (Figure 3, 4 h), bacterial cells began to collapse, and some of the folded cells remained rod-shaped. Further, after an 8-h treatment (Figure 3), 8 h the surfaces of the cells were more densely collapsed and wrinkled, and the cell morphology changed significantly. Moreover, after a 12-h treatment, no intact cells existed and only a fraction of cells could be observed, which indicated the whole lysis of cells.

### Transmission Electron Microscopy Observations

Transmission electron microscopy was also used to observe more details. As shown in Figure 4, in the control group, the cell membrane and cell wall were intact, and the cytoplasm was evenly dispersed. Compared with the control group, the cell morphology of the other three experimental groups changed significantly, and wrinkled surfaces (pointed by the red arrow, Figure 4), 4 h vacuoles inside the cytoplasm (pointed by the purple arrow, Figure 4), 4, 8, 12 h and the leakage of cytoplasm (pointed by the blue arrow, Figure 4), 8 and 12 h were clearly observed. Meanwhile, the boundaries of the cell membrane and cell wall became vague, and the distribution of cytoplasm became heterogeneous. Moreover, some extreme phenomena were also observed after the treatment of SMEO, such as the bare cell wall without cytoplasm inside it (pointed by the green arrow, Figure 4), 12 h and the individual cytoplasm without the protection of the cell wall (pointed by the blue arrow, Figure 4, 12 h).

### Flow Cytometry Analysis

Four fluorescent dyes (TO, PI, BOX, and EB) were used to evaluate several vital biological functions in *A. hydrophila* cells by flow cytometry analysis.

**FIGURE 5:**
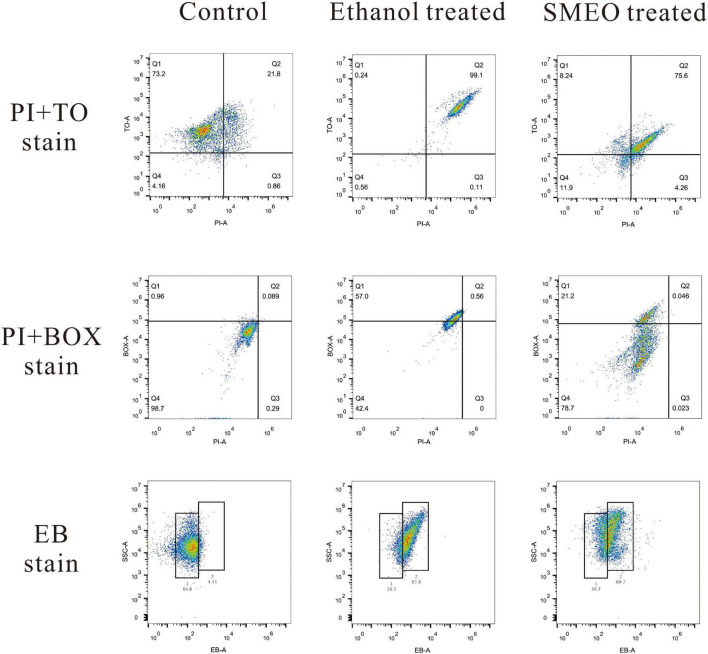
Fluorescence density plots of *A. hydrophila* treated with ethanol, SMEO, and PBS (Control), stained with propidium iodide (PI) and thiazole orange (TO), PI and bis-1,3-dibutylbutyric acid (BOX) and ethidium bromide (EB).

Membrane integrity was evaluated by double staining of TO and PI as shown in Figure 5 (the first row). In the control group, 73.2% of the cells were located in plot Q1 (TO + and PI–), which represented cells with intact cell membranes. Approximately 99.1% of the cells in the positive control group were located in plot Q2 (TO + and PI +), which represented cells with permeabilized cell membranes. In the SMEO-treated group, 75.6% of the cells were located in plot Q2 (TO + and PI +). Moreover, 11.9% of the cells were located in plot Q4 (TO- and PI-), which represented cells with damaged DNA or RNA.

Membrane potential was evaluated by double staining of BOX and PI as shown in Figure 5 (the second row). In the control group, 98.7% of the cells were located in plot Q4 (BOX- and PI-), which represented cells with polarized membranes. By contrast, in the SMEO-treated group and ethanol-treated group, 21.2% of the cells and 57.0% of the cells were located in plot Q1 (BOX + and PI-), respectively, which represented cells with depolarized membrane. Efflux activity was evaluated by EB staining as shown in Figure 5 (the third row). EB represented that the efflux pump functioned properly, while EB + meant the malfunction of the efflux pump. The percentage of EB + cells in the control and treated groups was 4.33 and 84.6%, and the percentage of EB- cells in these two groups was 60.2 and 36.3%.

## Discussion

Many species in *Citrus* L. are industrial crops and have a wide range of planting areas. In this study, SMEO was composed of 89 components, and the main chemical component was limonene (70.2252%). Meanwhile, other studies have exhibited that the content of limonene in the EO extracted from *Citrus unshiu* Marc. occupied a high percentage of content (Espina et al., 2011; Elmaci and Onoğur, 2012; Ioannou et al., 2012). Although not absolute, the extraction of *Citrus unshiu* Marc. by solid-phase micro-extraction showed that EO was not only composed of limonene but also linalool, γ-terpinene and β-elemene, p-cymene, and other components (Azam et al., 2013). The different composition of *Citrus unshiu* Marc. EO might be caused by different harvesting periods and extraction processes (Settanni et al., 2014).

As bacterial resistance has become a hindrance to antibiotics, the antibacterial activity of citrus leaf and peel extracts are prerequisites as potential alternatives to antibiotic drugs. This work indicated that SMEO has a bacteriostatic effect on *A. hydrophila*. Furthermore, studies have also indicated that *Citrus unshiu* Marc. EO has antibacterial activity against food-borne bacteria (Espina et al., 2011). In addition to *Citrus unshiu* Marc. EO, a variety of plant EOs exhibited the ability to inhibit the growth of *A. hydrophila* (Iturriaga et al., 2012; Ruiz-Navajas et al., 2012; Majolo et al., 2017). For example, *Thymus vulgaris*, *Eugenia caryophyllus*, and Tee Tree EO inhibited the growth of *A. hydrophila* (Assane et al., 2021). Screening of EOs of different plant-derived varieties indicated that 14 of them were found to be active against *A. hydrophila* (Kot et al., 2019). Therefore, SMEO has a great potential to reduce the use of antibiotics in aquaculture.

At present, there are limited details on the mechanism of how *Citrus unshiu* Marc. EO affects *A. hydrophila*. The cell membrane maintains the relative homeostasis of the intracellular environment to allow biochemical reactions to proceed normally in the cell. Therefore, the damage of the cell membrane is extremely detrimental to the survival of bacteria (Ali et al., 2016; Lu et al., 2016). Antimicrobial peptides from *Erythroculter ilishaeformis* killed bacteria by disrupting the integrity of *A. hydrophila* cell membranes (Chen et al., 2020). The mustard (*Brassica* spp.) EO affected the membrane permeability of *Escherichia coli* and *Salmonella typhi* (Turgis et al., 2009). In the present study, the absence of the cell protective function of the cell membrane was demonstrated by the changes in proteins and nucleic acids. Meanwhile, the results of microscopic observation also indicated that the cell membrane of *A. hydrophila* was severely deformed.

Flow cytometry, which facilitates the acquisition of data and the analysis of multiparameter rapidly, is an effective method widely used to evaluate antimicrobial activity and mechanism of action (Turgis et al., 2009; Wang et al., 2010). In this study, membrane integrity was evaluated by double staining of TO and PI. The results showed that, after the treatment of SMEO, the cell membrane of most of the *A. hydrophila* cells became unintegrated and permeable, with a certain degree of DNA or RNA damage, which was consistent with the phenomena observed by SEM and TEM. Membrane potential was evaluated by double staining of BOX and PI. Nearly all the cells in the control group had polarized membranes, but 21.2% of the cells in the treated groups had depolarized membranes. The loss of membrane potential might be explained by the change of ion concentrations inside and outside of the cell membrane, which were induced by the increase of cell permeability (Mirzoeva et al., 1997). These abovementioned results together with those observations of SEM and TEM indicated that the cell membrane should be an important target for SMEO and that the cell membrane was seriously damaged after SMEO treatment, finally resulting in the leakage of the cell content.

## Conclusion

Citrus EOs are applied in a variety of fields due to their various biological properties. In this study, SMEO was prepared and determined for composition. SMEO showed strong antibacterial activity against *A. hydrophila*. Intracellular material release, SEM, TEM, and flow cytometry analysis indicated that SMEO was capable of destabilizing the cell membrane. Therefore, the cell membrane was an important drug target for SMEO against *A. hydrophila*. Moreover, transcriptome and proteome technics will be used to explore the mechanisms at gene and protein levels.

## Data Availability Statement

The original contributions presented in the study are included in the article/supplementary material, further inquiries can be directed to the corresponding authors.

## Author Contributions

WZ: investigation and writing original draft preparation. KC and SJ: investigation. LY: visualization. TT: methodology. JG: validation and writing—review and editing. ZG: supervision, funding acquisition, and writing—review and editing. All authors contributed to the article and approved the submitted version.

## Conflict of Interest

The authors declare that the research was conducted in the absence of any commercial or financial relationships that could be construed as a potential conflict of interest.

## Publisher’s Note

All claims expressed in this article are solely those of the authors and do not necessarily represent those of their affiliated organizations, or those of the publisher, the editors and the reviewers. Any product that may be evaluated in this article, or claim that may be made by its manufacturer, is not guaranteed or endorsed by the publisher.
